# Validation and personality conditionings of the 3 × 2 achievement goal model in sport

**DOI:** 10.1038/s41598-024-52075-7

**Published:** 2024-01-18

**Authors:** Maciej Tomczak, Paweł Kleka, Ewa Tomczak-Łukaszewska, Łukasz Bojkowski, Małgorzata Walczak

**Affiliations:** 1 Department of Psychology, Poznan University of Physical Education, Poznań, Poland; 2 Faculty of Psychology and Cognitive Sciences, Adam Mickiewicz University, Poznań, Poland; 3 Faculty of English, Adam Mickiewicz University, Poznań, Poland

**Keywords:** Psychology, Human behaviour

## Abstract

Achievement goal models have been successfully applied in sport. In recent years, a framework that has emerged in this area is the 3 × 2 approach, being a natural evolution of years of research into the issue of achievement goals. Nevertheless, it is essential to further validate the abovementioned approach and explore its psychosocial context. Hence, the purpose of this study was to validate the latest 3 × 2 achievement goal model among high-performance and recreational athletes using the Polish version of the 3 × 2 Achievement Goal Questionnaire for Sport (3 × 2 AGQ-S), and to determine the relationship between personality traits and achievement goals of athletes. The study included 413 athletes, with M = 20.62 and SD = 2.72. The 3 × 2 AGQ-S was used to assess achievement goals, the Big Five personality traits were assessed using the IPIP-BFM-20 questionnaire. The model of the Polish version of the 3 × 2 AGQ-S achieved a satisfactory fit to the data (CFI = 0.940, TLI = 0.923, RMSEA = 0.086, SRMR = 0.061). Cronbach’s alphas for the subscales were: 0.77–0.96. High-performance athletes obtained higher achievement goals in task and self subscales than recreational athletes. Personality traits explained no more than 3% of variance of achievement goals in sport. Research involving the Polish version of the 3 × 2 AGQ-S supports the validity of the 3 × 2 model in sport among high-performance and recreational athletes. Nevertheless, the small relationships between the personality traits and achievement goals prompt searching for other psychosocial determinants of goals in sport.

## Introduction

Achievement-related motivational factors constitute the most relevant and common motivational constructs involved in sport, and successively developed approaches to achievement goal models fall within this area. The originally established framework was the so-called dichotomous model, which was first used in the school environment and only later in sport, distinguishing two possible goal orientations, i.e., the task (mastery) and the ego (performance)^1–4^. In the light of Achievement Goal Theory, athletes characterized by a high task orientation are learning-driven, and evaluate their competence by effective task performance, whereas athletes with a high ego orientation assess their competence by comparing their level of task performance with that of others^4,5^. Subsequently, the ego-performance goals were extended to include the “approach”—“avoidance” components^6^, which led to the development of a trichotomous model used in school, work and sport context^7,8^. The goals of athletes with a high level of a performance-approach consisted in performing better than others, while athletes with a high level of performance-avoidance aimed to avoid inferior performance in comparison with others^8^. Subsequently, the concept of approach-avoidance was also included in the mastery construct, resulting in the so-called 2 × 2 model^9–11^, which is frequently applied in sport, as well as in the field of physical activity in general^12–15^. According to this view, athletes with a high mastery-approach stipulate goals focused on efficient task performance and improvement, whereas the goals of athletes with a high mastery-avoidance consist in avoiding inefficient task performance and avoiding performing worse than they did before^7,8^.

Subsequently, the 2 × 2 model was modified to a 3 × 2 model by extracting—from the overall mastery construct—the following factors: task-based (reference to the absolute requirements of the task) and self-based (reference to one’s own task performance)^8,16^. Therefore, currently the model includes six subscales, i.e. task-approach, task-avoidance, self-approach, self-avoidance, other-approach, and other-avoidance. Athletes with a high task-approach tend to be focused on efficient task performance and good results, whereas athletes with a high task-avoidance avoid poor task performance and poor results. In contrast, athletes with a high self-approach aim to perform better and be more efficient than they were before, whilst athletes characterized by self-avoidance strive to avoid poorer task performance and being less efficient than before. Individuals with a high other-approach adopt the goal of performing better and being more effective than others. Conversely, the goal of the individuals with a high other-avoidance is to avoid performing their task more poorly and being less effective than others^8,16^. This latest approach to achievement goal models (3 × 2 model) has only recently been used in sport^8^. Although it has been evaluated in certain cultural contexts, it requires further validation through conducting analyses in different groups of athletes and evaluation of its relationships with various variables which are relevant to functioning in sport.

Taking into account the abovepresented content, the aim of our research was to validate the 3 × 2 model in sport in the Polish cultural context (validation of the Polish version of the 3 × 2 AGQ-S), and to determine the relationship between the Big Five personality traits and achievement goals in sport. Validation of the new tool will expand the diagnostic options in the field of motivation in sport in Poland, and will allow further scientific research involving the latest achievement goal model (3 × 2 model). In addition, the conducted validation includes both high-performance athletes and recreational athletes. A comparison of the level of goal achievement in these groups of athletes will also be performed, and in order to assess the similarity of the use of the 3 × 2 AGQ-S, the invariance of measurement in these groups will be examined. This comparison will considerably support the validation of the tool, and has been justified by previous research results indicating significant differences in various components of motivation to undertake sport activities between professional and recreational athletes^17^, as well as in goal orientation. For example, Tomczak et al.^18^ demonstrated higher levels of ego orientation (POSQ scale) in high-performance athletes than in recreational athletes. In the case of the 3 × 2 model, it may also be assumed that high-performance athletes will be characterized by higher levels of a newly emerging factor in this view, i.e., task-approach. This type of goals (in the 3 x 2 AGQ-S) is related to a focus on high levels of performance and good results, which appears to be more characteristic of professional athletes^8^. Our study will also compare achievement goals according to gender. In studies including the Polish version of the POSQ scale and the GOEM scale, men were characterized by higher ego levels than women^18,19^.

Regarding the conditionings of achievement goals, personality factors play an important role. To the best of our knowledge, the relationship between personality traits and achievement orientations/goals from the latest 3 × 2 model has not yet been determined, and to date no such research has been undertaken in the sport domain. Hence, the next goal of our research was to assess these relationships. The significance of personality traits in relation to achievement motivation has been related to searching for more basic predispositions for the development of the specific goal orientation. It is of note that Payne, Youngcourt and Beaubien^20^ refer to personality traits as “antecedents” for the development of goal orientation. The underlying importance of personality traits has been emphasized, indicating that goal orientations as cognitive constructs may have a regulatory meaning, therefore they also specify general personality tendencies^21,22^. Similarly, Sorić et al.^23^ indicate that goal orientations may have the status of self-regulated learning components and act as mediators between personality and achievement. In turn, Zweig and Webster^24^ emphasize that goal orientations, as a distinct construct for personality, may mediate the relationship between personality traits and performance intention.

Previous research confirms that personality traits from the Big Five model^25^ have significant relationships with achievement orientations/goals. For instance, a meta-analysis^26^ of the relationships between Big Five personality traits and achievement orientations/goals from the 2 × 2 model in primarily school and work environments indicated that conscientiousness is generally positively related with a mastery-approach and performance-approach, and negatively related with performance-avoidance. Such relationships seem reasonable, since the components of conscientiousness include achievement motivation, self-discipline and dutifulness^27^, which are also relatively close to the mastery orientation associated with efficient execution of tasks. In addition, conscientiousness components associated with an achievement orientation may favor setting goals for outperforming others (performance-approach) as opposed to a performance-avoidance orientation^28^. In the mentioned meta-analysis^26^, it has also been shown that extraversion, openness and agreeableness are, in general, positively related to a mastery-approach, and negatively related to performance-avoidance. The same directions of correlation for the referred personality traits with learning orientation (mastery factor) were obtained by Mahlamäki et al.^29^. By analyzing the content of the constructs, it can be concluded that indicators and correlates of extraversion include a tendency to be active, optimistic, energetic, showing positive emotionality and the need for stimulation^27^, which may also tend to favor orientation towards active action through goal-setting linked to a learning-oriented mindset, i.e. mastery-approach^30^, as opposed to a performance-avoidance mindset which is inferior to others. Willingness to cooperate (the agreeableness component) and attitudes toward novelty and the creative attitude, openness and cognitive curiosity (indicators of openness to experience)^31^ may tend to favor development and skill improvement attitudes related with a high mastery-approach and low performance-avoidance^24^. Additionally, negative and weaker relationships were related to agreeableness and performance-approach indicating the predictable direction of the relationship: cooperative orientation (high agreeableness) does not favor setting goals involving being better than others. In turn, neuroticism has generally been shown to be negatively related with mastery-approach, and positively with a performance-approach and performance-avoidance^32^. Indicators and correlates of neuroticism are anxiety-like behaviors, instability, depressiveness, and a tendency to experience negative emotions^31^, which may tend to favor defensive/unique behaviors and performance-avoidant ways of achieving goals as opposed to orientation to efficient execution of tasks: the mastery-approach^33^. In contrast, lower emotional stability and less behavioral control associated with neuroticism may be associated with an excessively competitive (performance-approach) orientation. These relationships are also supported by the research by Miller et al.^34^, who found positive relationships of neuroticism with performance-approach and avoidance. In contrast, fewer studies have been reported for the mastery-avoidance factor (e.g. only two studies with different results in the aforementioned meta-analysis).

Thus, in the light of the research findings and rationale presented earlier, we assume that personality traits will be related with achievement goals in sport. More specifically, we hypothesize that personality traits such as conscientiousness, intellect (openness), extraversion, agreeableness and emotional stability (the inverse of neuroticism) will positively correlate with both task-approach and self-approach (components of mastery); additionally, we assume that emotional stability and agreeableness will negatively correlate with other-approach (performance-approach); we also hypothesize that conscientiousness will positively correlate with other-approach; additionally, we assume that traits such as conscientiousness, intellect, extraversion, agreeableness and emotional stability will negatively correlate with other-avoidance (performance-avoidance).

In summary, in the light of our knowledge, current studies contribute certain new insights in terms of validation and the relationships between the variables examined in relation to the 3 × 2 achievements goals model in sport. An important novelty of the study is to test the measurement invariance of the 3 × 2 AGQ-S in groups of high-performance and recreational athletes, and to compare the level of achievement goals in these groups taking into account gender. Another new feature is the assessment of the reliability of the test items and subscales of the 3 × 2 AGQ-S by analyzing the information curves from the Item Response Theory approach. Finally, an important novelty of the study is also the identification of relationships between personality traits and achievement goals from the new 3 × 2 model in sport in total and among both high-performance and recreational athletes. Previously, such relationships were determined in relation to older achievement goal models. The presentation of these relationships with taking into account the recently developed 3 × 2 model will significantly expand the area of personality determinants of achievement goals in sport.

## Methods

### Participants

The study included 413 athletes with age M = 20.62, SD = 2.72. They comprise 273 men and 140 women. A group of 240 athletes declared that they practiced sports professionally, systematically participated in sport competitions and train mainly to achieve the best sport results. They took part in national-level sport competitions, among others. Average years of training in high-performance athletes was M = 9.77, SD = 3.70. The other group consisted of 173 individuals who declared that they practiced sports recreationally—they participated in various competitions and sport tournaments, although they trained mainly for health and to improve their physical fitness. Football, volleyball, dance, basketball, taekwondo, swimming, and running were the most represented sport disciplines among the participants.

### Instruments

The 3 × 2 Achievement Goal Questionnaire for Sport (3 × 2 AGQ-S) by Mascret et al.^8^ was used to test goal achievements. The questionnaire consisted of 18 statements, and the respondents’ task was to rate on a scale from 1 to 7 how much each statement applied to them. The questionnaire contained 6 subscales (3 statements for each), i.e., task-approach, task-avoidance, self-approach, self-avoidance, other-approach, other-avoidance. First, a language expert translated the scale into Polish. Then another language expert back-translated the scale into English. A group of experts consisting of three sport psychologists and a translator established the final Polish version of the scale. At the outset, the subjects were asked to raise any doubts they had about their understanding of the statements. Yet, no doubts were raised.

The IPIP-BFM-20 questionnaire was used to determine the personality traits from the Big Five model. It consisted of 20 test items, with four test items for each of the five personality traits (emotional stability: α = 0.76, extraversion: α = 0.83, agreeableness: α = 0.68, conscientiousness: α = 0.73, intellect: α = 0.70). The respondents’ task was to assess how much each statement applies to them on a 1–5 scale^35^.

### Procedure

The participants were first informed that their participation in the study was voluntary and that they could withdraw from the study at any time. They were also asked if they had any health contraindications, physical and mental state conditions at the time of the study, or any other obstacles that would hinder their participation or prevent them from taking part in the study. None of the participants reported ill health or contraindications to taking part in the study. The study had no time limit. The survey was questionnaire-based and fully anonymous. The description of the research was reviewed by the Bioethics Committee at Poznan University of Medical Sciences (Poland), who issued a statement that the research did not bear the features of a medical experiment and in accordance with the Polish law and GCP was not subject to the opinion of the Bioethics Committee (Statement No. KB-273/22). The Bioethics Committee issues their opinions primary on the basis of the Act of 5 December 1996 on professions of doctor and dentist (including subsequent amendments). In accordance with the recommendations of the Bioethics Committee, the study instructions contained a statement that taking part in the study and handing in the completed questionnaire would be considered as a participant’s informed consent to take part in the study. The study was conducted in accordance with the Declaration of Helsinki. The study is part of a large-scale research project on measurement and psychosocial correlates of goal orientation in high performance and recreational athletes with respect to physical activity they undertake.

### Statistical analysis

Confirmatory factor analysis was used to assess factor validity. Due to fact that the distribution of the data deviated from a normal distribution, the Satorra-Bentler^36^ correction was applied^37,38^. First, the theoretical model of the 3 × 2 AGQ-S was tested. CFI and TLI above 0.90 were assumed to indicate satisfactory values of model fit to the data, and 0.95 and above indicated a very good fit. In contrast, with regard to SRMR and RMSEA, the values below 0.08 were considered satisfactory, while RMSEA values between 0.08 and 0.10 were considered a mediocre fit and above 0.10 were considered a poor fit^39–42^. Following the authors of the original version of the questionnaire^8^, the fit of alternative models to the data was also verified in order to compare it with the original 3 × 2 model. Alternative models with the following arrangement of factors were tested: (1) a model where the task and self with the same valence were placed on combined factors, and the other-subscales were on their hypothesized factors (2 × 2 model); (2) a model where all items from the task and the self-subscales were placed on one factor, and the other-items on their factors (separate factors for the other-approach and other-avoidance—the trichotomous model); (3) a model where all items from the task and the self-subscales were on one factor, and all items from the other-subscales were on the second factor (the dichotomous model); (4) a model where items from the task-approach and the task-avoidance subscales were on one factor, and all the remaining items were on their factors (Tap/Tav model); (5) a model where items from the self-approach and the self-avoidance subscales were on one factor, and all the remaining items are on their factors (Sap/Sav model); (6) a model where items from the other-approach and the other-avoidance subscales were on one factor, and all the remaining items were on their factors (Oap/Oav model); (7) a model where all items from the approach subscales were placed on one factor, and items from the avoidance subscales were on their factors (approach model); (8) a model where all items from the avoidance subscales were placed on a single factor, and items from the approach subscales were placed on their factors (avoidance model); (9) a model where items that share competence definition were placed on combined factors (three factors: the task, self, other—definition model); (10) a model where two factors were identified based on the valence of approach-avoidance (valence model).

We also analyzed the invariance of measurement with the 3 × 2 AGQ-S scale for the 3 × 2 model among the high-performance and recreational athletes, as well as among men and women. The configural was verified first, followed by the metric (factor loadings in the analyzed groups were fixed), scalar (intercepts in the groups were fixed) and strict (residual values in the groups were fixed). It was assumed that a decrease in CFI values above 0.01 and an increase in RMSEA above 0.015 indicated significant differences between the study groups^43^.

Cronbach’s alpha coefficient was used to assess the reliability of the scale. In addition, the quality of subscales and items was assessed by analyzing information curves from the Item Response Theory—the generalized partial credit model. Highly populated information curves were indicative of the large amount of information provided by the measurement of a trait in a specific area and, thus, less measurement error and greater precision of a subscale or item in that area^44,45^.

In addition, as part of investigating validity, a comparison using two-way analysis of variance was made between individuals of different gender and with different levels of sport participation (high-performance, recreational) in terms of the level of achievement goals in sport as measured by the 3 × 2 AGQ-S questionnaire. To assess individual relationships between the personality traits and achievement goals r-Pearson was used. Multiple regression analysis was used to assess the combined contribution of the personality traits to achievement goals in sport. The analysis was performed using R environment and Statistica software (ver.13.3).

## Results

### Construct validity of the 3 × 2 AGQ-S

**Figure 1:**
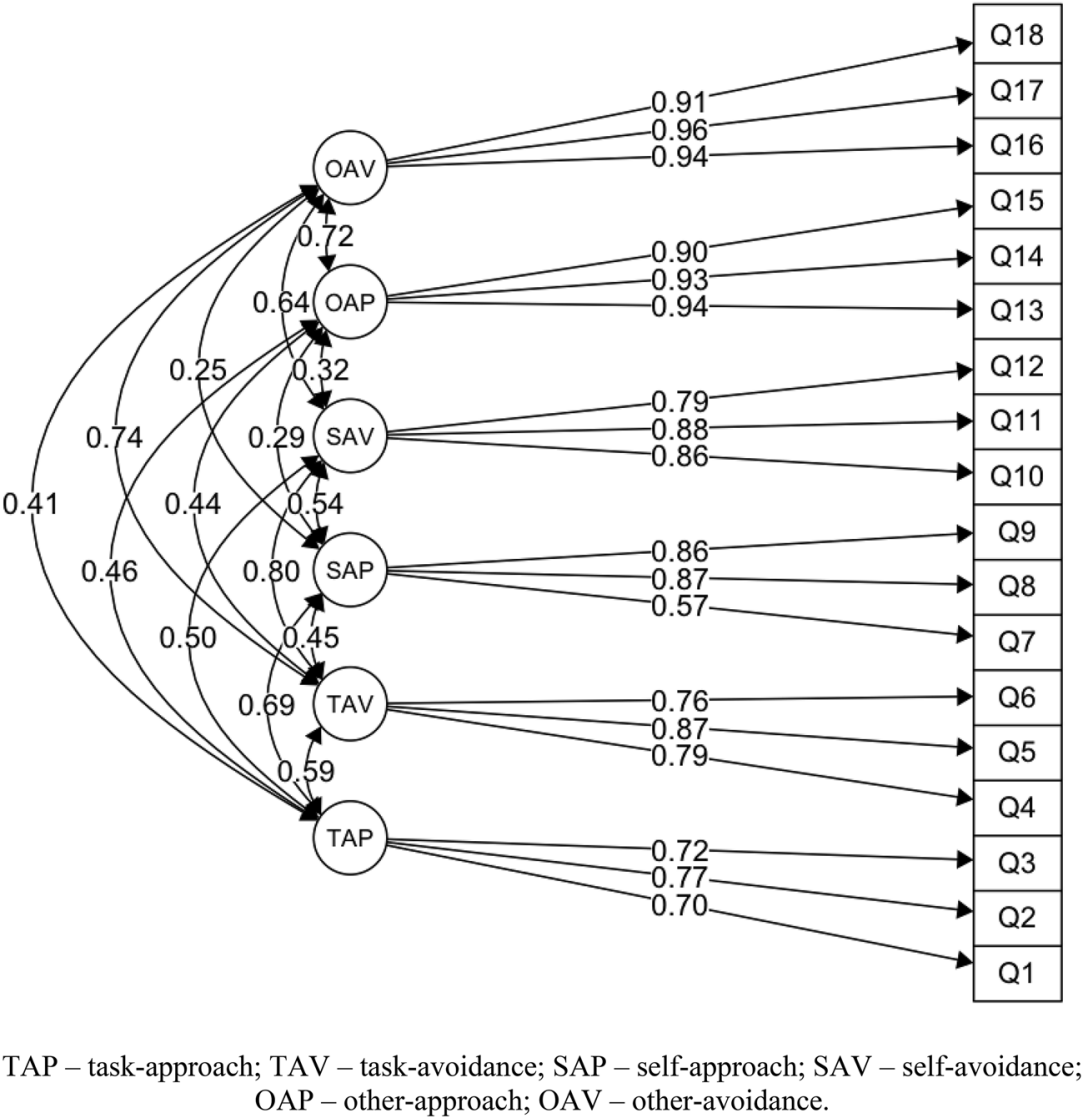
Model 1—confirmatory factor analysis (CFA) for the 3 × 2 model.

Model No. 1 (Fig. 1) estimated in line with the theoretical assumptions of the 3 × 2 achievement goals in sport model, obtained the best (compared to alternative models) and a generally good fit to the data in terms of CFI, TLI, SRMR indexes. Only the RMSEA value was slightly too high. The other models, which were estimated according to other theoretical assumptions, did not provide a good fit to the data (Table 1).

**Table 1:** Fitting the original 3 × 2 achievement goal model (Model 1) and alternative models to the data.

Model	X^2^ (df)	CFI	TLI	RMSEA	SRMR
3 × 2 (original)	354.374 (120)	0.940	0.923	0.086	0.061
2 × 2	535.842 (129)	0.894	0.874	0.110	0.069
Trichotomous	827.534 (132)	0.820	0.791	0.142	0.096
Dichotomous	1200.184 (134)	0.709	0.667	0.179	0.107
Task ap/av	537.229 (125)	0.895	0.872	0.111	0.088
Self ap/av	599.884 (125)	0.879	0.852	0.119	0.080
Other ap/av	825.373(125)	0.816	0.775	0.147	0.087
Approach	961.062 (129)	0.788	0.748	0.156	0.165
Avoidance	911.996 (129)	0.801	0.764	0.151	0.121
Definition	1130.343 (132)	0.731	0.688	0.174	0.105
Valence	1412.962 (134)	0.672	0.625	0.190	0.170

The factor loadings for model 1 (original) are presented in Fig. 1.

The estimated models under invariance analysis fit the data relatively well. The decreases in CFI are less than 0.01 and the RMSEA values do not increase above 0.015 in any case, hence the assumption of measurement invariance in the study groups (women–men, high-performance athletes–recreational athletes) can be taken as confirmed (Table 2).

**Table 2:** Measurement invariance of the 3 × 2 AGQ-S scale by gender and level of sport participation.

	CFI	RMSEA	ΔCFI	ΔRMSEA	
Gender (males vs. females)					
Configural	0.928	0.095			
Metric	0.929	0.092	0.001	0.003	
Scalar	0.929	0.090	0.000	0.002	
Strict	0.926	0.089	0.003	0.001	
Level of participation (high-performance vs. recreational)					
Configural	0.941	0.084			
Metric	0.940	0.083	0.001	0.001	
Scalar	0.937	0.083	0.003	0.000	
Strict	0.932	0.084	0.005	0.001	

### Reliability indexes—internal consistency

The following Cronbach’s alpha values were obtained: task-approach: α = 0.77, task-avoidance: α = 0.85, self-approach: α = 0.78, self-avoidance: α = 0.88, other-approach: α = 0.94, other-avoidance: α = 0.96.

### Information curves for the 3 × 2 AGQ-S questionnaire—The IRT model

**Figure 2:**
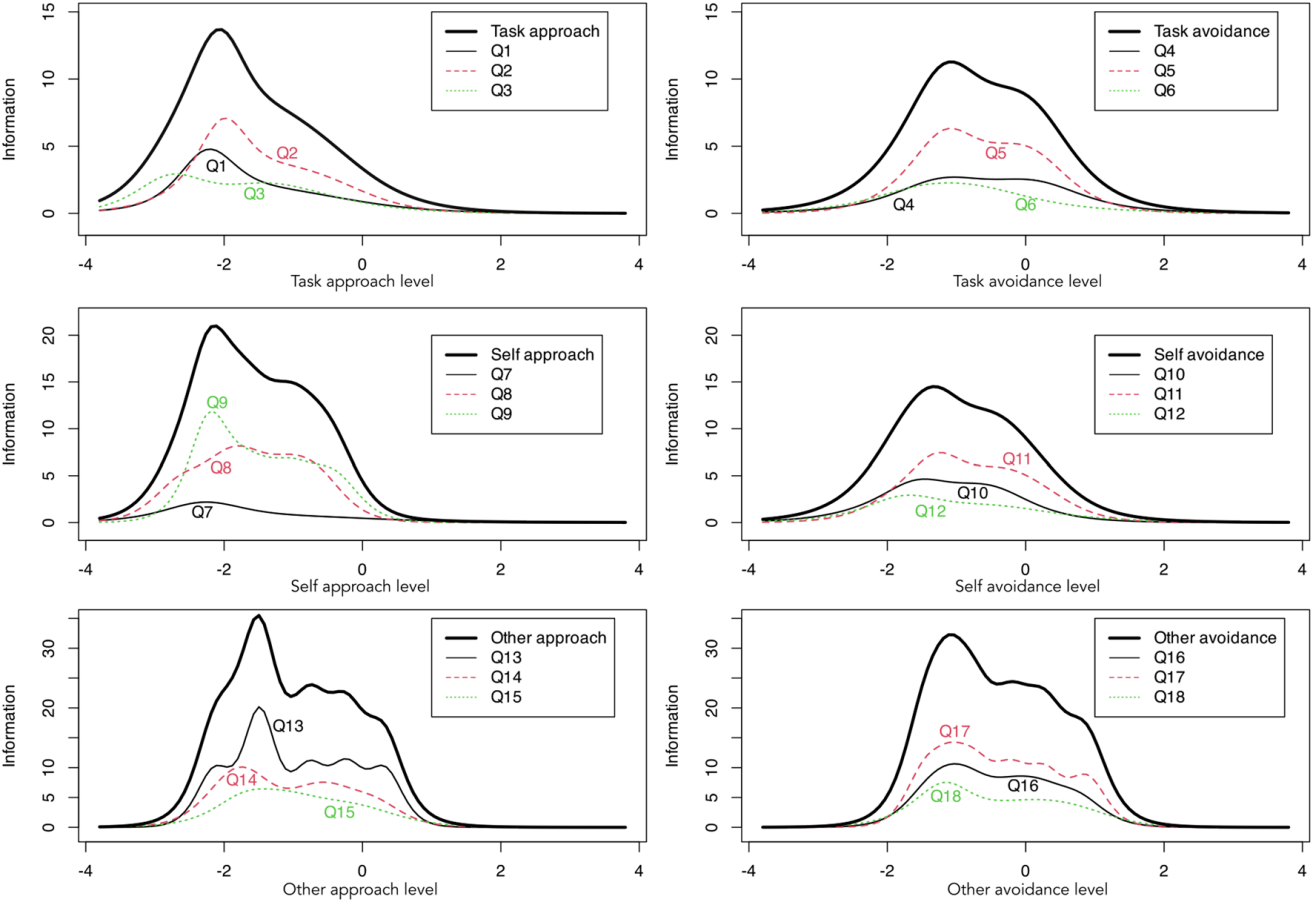
Information curves for subscales (thick line) and items of the 3 × 2 AGQ-S questionnaire.

The graphs presenting the information curves indicate that, in general, more information was obtained for the scores of low and medium subscales, as well as items of the 3 × 2 AGQ-S questionnaire. In particular, more information for lower trait values was obtained for the task-approach and self-approach subscales. The other subscales and items also contained a lot of information from the area of medium scores, and less from the high scores (Fig. 2).

### Gender, level of participation and achievement goals in sport

The main effect of level of sport participation was observed for the task-approach: F(1, 409) = 22.31, p < 0.001, η_p_^2^ = 0.052. High-performance athletes showed higher values than recreational athletes (Table 3). However, no main effect of gender (F(1, 409) = 1.29, p > 0.05), as well as and no interaction of gender*level of sport participation were found (F(1, 409) = 0.23, p > 0.05). The main effect of level of sport participation was obtained for task-avoidance: F(1, 409) = 12.26, p < 0.001, η_p_^2^ = 0.029. High-performance athletes presented higher values than recreational athletes (Table 3). Additionally, no main effect of gender (F(1, 409) = 0.09, p > 0.05) and no interaction of gender*level of participation were observed (F(1, 409) = 0.09, p > 0.05).

**Table 3:** Level of achievement goals in groups of high-performance and recreational athletes, and in groups of women and men.

N = 413		N	Tap M(SD)	Tav M(SD)	Sap M(SD)	Sav M(SD)	Oap M(SD)	Oav M(SD)
HP	–	240	6.44 (0.74)	5.36 (1.45)	6.47 (0.74)	5.71 (1.39)	5.37 (1.54)	5.02 (1.72)
R	–	173	6.04 (0.91)	4.82 (1.59)	6.14 (0.94)	5.37 (1.44)	4.99 (1.66)	4.71 (1.83)
F	–	140	6.20 (0.79)	5.15 (1.49)	6.38 (0.82)	5.67 (1.17)	4.93 (1.58)	4.85 (1.65)
M	–	273	6.31 (0.86)	5.12 (1.56)	6.31 (0.86)	5.52 (1.53)	5.35 (1.59)	4.91 (1.84)
HP	F	77	6.40 (0.62)	5.42 (1.38)	6.50 (0.67)	5.85 (1.06)	4.97 (1.55)	4.93 (1.52)
R	F	63	5.95 (0.91)	4.81 (1.56)	6.22 (0.95)	5.44 (1.27)	4.87 (1.63)	4.76 (1.81)
HP	M	163	6.45 (0.80)	5.33 (1.49)	6.45 (0.78)	5.65 (1.52)	5.55 (1.51)	5.06 (1.81)
R	M	110	6.09 (0.91)	4.82 (1.62)	6.10 (0.94)	5.33 (1.53)	5.06 (1.67)	4.68 (1.85)

Subscales from the 3 × 2 AGQ-S model: *Tap* task-approach; *Tav* task-avoidance; *Sap* self-approach; *Sav* self-avoidance; *Oap* other-approach; *Oav* other-avoidance.

*HP* high-performance athletes; *R* recreational athletes;* F* female; *M* male.

The main effect of the level of sport participation was noted for the self-approach: F(1, 409) = 13.13, p < 0.001, η_p_^2^ = 0.031. High-performance athletes differed from recreational athletes (Table 3). There was no significant effect of gender (F(1, 409) = 0.95, p > 0.05) and no interaction of gender*level of participation (F(1, 409) = 0.18, p > 0.05). In terms of self-avoidance, the main effect of sport participation level was noted: F(1, 409) = 6.11, p < 0.05, η_p_^2^ = 0.015. High-performance athletes showed higher values than recreational athletes (Table 3). No main effect of gender (F(1, 409) = 1.12, p > 0.05) and no interaction of gender*level of participation were found (F(1, 409) = 0.11, p > 0.05).

The main effect of gender was noted for the other-approach: F(1, 409) = 5.39, p < 0.05, η_p_^2^ = 0.013. Men presented higher values than women (Table 3). There was no main effect of the level of participation (F(1, 409) = 3.31, p > 0.05) and no interaction of gender*level of participation (F(1, 409) = 1.39, p > 0.05). In terms of the other-avoidance, there was no significant effect of gender (F(1, 409) = 0.02, p > 0.05), level of participation (F(1, 409) = 2.18, p > 0.05) or interaction gender*level of participation (F(1, 409) = 0.32, p > 0.05).

### Relationship between personality traits and achievement goals in sport

Small positive correlations of extraversion with the task-approach, task-avoidance, and other-approach were observed. In addition, there were small positive correlations of conscientiousness with the task-approach, self-approach and task-avoidance. Emotional stability was weakly positively related with the task-approach. In turn, intellect was weakly positively correlated with the self-approach and other-approach (Table 4).

**Table 4:** Relationships between personality traits and achievement goals in sport for the whole group.

Variables	N = 413 (r-Pearson)					
	Tap	Tav	Sap	Sav	Oap	Oav
EXT	0.13**	0.10*	0.07	0.08	0.10*	0.08
AGR	0.03	0.05	0.02	0.04	− 0.08	− 0.04
CON	0.16**	0.10*	0.15**	0.09	0.03	0.02
ES	0.10*	− 0.06	0.02	− 0.06	0.01	− 0.06
IN	0.09	− 0.03	0.12*	− 0.01	0.13**	0.05

Subscales from the 3 × 2 AGQ-S model: *Tap* task-approach; *Tav* task-avoidance; *Sap* self-approach; *Sav* self-avoidance; *Oap* other-approach; *Oav* other-avoidance. The Big Five personality traits (IPIP-BFM-20): *EXT* extraversion; *AGR* agreeableness; *CON* conscientiousness; *ES* emotional stability; *IN* intellect.

*For *p* < 0.05; ** for *p* < 0.01.

In line with small correlations, regression analysis showed that personality variables accounted for a small proportion of achievement goals in sport**—**determination coefficients did not exceed 3%, and the personality traits as predictors demonstrated relatively small regression coefficients (Table 5).

**Table 5:** Summary of regression analyses with personality traits as predictors and achievement goals as dependent variables.

Predictors	N = 413, regression analysis: standardized beta coefficients					
	Tap	Tav	Sap	Sav	Oap	Oav
EXT	0.11*	0.13*	0.06	0.10	0.10*	0.10
AGR	− 0.01	0.02	− 0.01	0.01	− 0.12*	− 0.07
CON	0.15**	0.12*	0.16**	0.11*	0.04	0.04
ES	0.04	− 0.09	− 0.04	− 0.10	− 0.05	− 0.10
IN	0.05	− 0.05	0.11*	− 0.01	0.13*	0.06
R^2^ =	0.03	0.02	0.03	0.01	0.03	0.01
F(5, 407) =	3.96**	2.79*	3.32**	1.99	3.20**	1.71

Subscales from the 3 × 2 AGQ-S model: *Tap* task-approach; *Tav* task-avoidance; *Sap* self-approach; *Sav* self-avoidance; *Oap* other-approach; *Oav* other-avoidance. The Big Five personality traits (IPIP-BFM-20): *EXT* extraversion; *AGR* agreeableness; *CON* conscientiousness; *ES* emotional stability; *IN* intellect.

*For *p* < 0.05; ** for *p* < 0.01.

Similarly to when the group was viewed as a whole, the relationships between the personality traits and achievement goals in separate groups (high-performance athletes, recreational athletes, women, men) were relatively small or failed to achieve statistical significance with a p-value at 0.05 (Table 6).

**Table 6:** Relationships between personality traits and achievement goals in sport in groups of high-performance and recreational athletes, and in groups of women and men.

Variables	High-performance athletes N = 240 (r-Pearson)						Recreational athletes N = 173 (r-Pearson)					
	Tap	Tav	Sap	Sav	Oap	Oav	Tap	Tav	Sap	Sav	Oap	Oav
EXT	0.14*	0.09	0.12	0.07	0.13*	0.12	0.11	0.11	0.02	0.08	0.07	0.03
AGR	0.01	0.13*	0.05	0.11	− 0.01	0.02	0.04	− 0.07	− 0.02	− 0.06	− 0.16*	− 0.12
CON	0.18**	0.12	0.15*	0.11	− 0.04	0.03	0.11	0.05	0.13	0.06	0.09	− 0.01
ES	0.04	− 0.15*	0.01	− 0.08	0.01	− 0.06	0.18*	0.06	0.04	− 0.04	0.02	− 0.05
IN	0.04	− 0.01	0.12	0.04	0.13*	0.05	0.13	− 0.08	0.10	− 0.06	0.13	0.04

Variables	Women N = 140 (r-Pearson)						Men N = 273 (r-Pearson)					
	Tap	Tav	Sap	Sav	Oap	Oav	Tap	Tav	Sap	Sav	Oap	Oav
EXT	0.06	0.07	0.03	0.15	0.13	0.07	0.16**	0.11	0.08	0.04	0.10	0.09
AGR	0.02	0.02	− 0.13	0.11	− 0.08	0.01	0.05	0.06	0.08	0.01	− 0.04	− 0.06
CON	0.15	0.03	0.10	0.03	− 0.01	− 0.09	0.16**	0.14*	0.18**	0.12*	0.05	0.07
ES	0.13	− 0.05	0.09	− 0.06	− 0.02	− 0.07	0.06	− 0.06	0.01	− 0.04	− 0.03	− 0.06
IN	0.23*	0.10	0.14	0.12	0.19*	0.17*	0.01	− 0.10	0.11	− 0.05	0.08	− 0.01

Subscales from the 3 × 2 AGQ-S model: *Tap* task-approach; *Tav* task-avoidance; *Sap* self-approach; *Sav* self-avoidance; *Oap* other-approach; *Oav* other-avoidance. The Big Five personality traits (IPIP-BFM-20): *EXT* extraversion; *AGR* agreeableness; *CON* conscientiousness; *ES* emotional Stability; *IN* intellect.

*For *p* < 0.05; ** for *p* < 0.01.

## Discussion

In order to study the validity of the 3 × 2 Achievement Goal Questionnaire for Sport, the authors analyzed its factorial validity, and the relationship of the results from the 3 × 2 AGQ-S with athletes’ level of sport participation. In terms of factor validity, an overall satisfactory fit of the 3 × 2 model to the empirical data was demonstrated based on the CFI, TLI (values above 0.90) and SRMR (nearly 0.06). Nevertheless, the RMSEA value was slightly too high as it was above 0.08, yet below 0.10 (the value considered unacceptable). The fit of the model to the data, however, was poorer than the fit obtained by the authors of the original version of the questionnaire^8^ and slightly worse than the fit values obtained among mainly Chinese athletes by Wang et al.^46^, who adapted the questionnaire directly from the classroom environment to sport. In contrast, the fit indexes of the Polish version were mostly better than those of the Brazilian version^47^. The validity of the Polish version of the questionnaire was also confirmed by the best fit of the 3 × 2 model compared to the competing models taking into account the various assumptions described in the Methods Section (e.g., the 2 × 2 model, the trichotomous and dichotomous model). This result was consistent with the findings of the authors of the original version of the scale^8^. High values of internal consistency indexes (Cronbach’s alpha) were also obtained. They were mostly similar to the results obtained by the authors of the original version of the scale^8^. In addition, the quality of the subscales and items was also assessed by means of analyzing the information curves from the IRT model. In general, the subscales and test items provide more information and, thus, more precise measurement in the lower and middle areas of the traits studied. In particular, the task-approach and self-approach subscales show higher precision for low values and lower precision for medium and high values of the traits. This result was consistent with the previous analysis of information curves for the task subscale from the Polish versions of the POSQ^18^, TEOSQ^48^ and GOEM^19^ questionnaires.

Measurement invariance was also analyzed in groups of men and women, as well as in groups of high-performance and recreational athletes. There were no large differences in the values of model fit indexes to the data for different types of invariance. Therefore, it can be concluded that women use the 3 × 2 AGQ-S in a similar way to men, and high-performance athletes use it similarly to recreational athletes. It was also demonstrated that high-performance athletes were characterized by higher levels of task-approach, task-avoidance, self-approach and self-avoidance than athletes competing at the recreational level. The results for the task subscales are particularly substantiated, as competitive sports are associated with a greater focus on effectiveness and good performance, which is reflected in the content of these subscales. However, the differences (effects) obtained were not large (based on effect size), and the mean values obtained by recreational athletes on these scales were not low either. This may be related to the fact that the study included recreational athletes who engaged in physical activity with rivalry, which in part may share the characteristics of a competitive sport activity. In addition, men obtained higher scores than women on the other-approach scale, which also supports the validity of the tool. This result is consistent with the results obtained for the Polish versions of the GOEM^19^ and POSQ^18^ scales. Furthermore, this appears to be justified, as it has been emphasized that men are more competitive than women, and are more inclined to display their abilities^49^, which may be related to both evolutionary and social aspects^19^.

Another objective of the study was also to evaluate the relationships between the Big Five personality traits model and the achievement goals from the 3 × 2 model in sport. The observed relationships were small. Hence, despite certain differences depending on the separate groups, the correlations slightly differed. However, it is possible to relate the obtained results to previous research findings in terms of the directions of the obtained correlations. Extraversion, conscientiousness and emotional stability were positively related with the task-approach. In addition, conscientiousness and intellect were also positively related to self-approach. The directions of these relationships were quite consistent with our assumptions and the results presented by McCabe et al.^26^ for the 2 × 2 model. The same directions of correlation of the mentioned personality traits with learning orientation (mastery) were obtained by Kaspi-Baruch^50^ and Mahlamäki et al.^29^. Additionally, in the case of our participants, extraversion and conscientiousness were positively related with task-avoidance. Due to the smaller number of studies in the presented meta-analysis devoted to the mastery-avoidance factor (divided into task- and self-avoidance in the 3 × 2 model), in this case the comparison of the results is less reliable. However, partially similar relationships were obtained by Chen and Zhang^33^, who showed positive relationships of mastery-avoidance with extraversion, conscientiousness and openness. Additionally, Corker et al.^28^ and Asanjarani et al.^51^ reported positive correlations between mastery-avoidance and neuroticism. In addition, there was no correlation in our study of agreeableness and intellect with the task-approach, as well as no correlations of emotional stability, extraversion and agreeableness with the self-approach, which is not in line with our assumptions. Yet, there were sometimes similar results, e.g. Wang and Erdheim^22^ did not show significant relationships of agreeableness, openness and neuroticism with the mastery (learning) factor, and Hendriks and Payne^52^ did not obtain significant correlations of the learning orientation (mastery) factor with agreeableness and neuroticism. On another note, maybe the obtained results stem partly from the fact that the 3 × 2 model splits the mastery factor into two components (task and self) and/or that the present study was performed in a sport context as opposed to the studies presented in the meta-analysis, which were conducted primarily in school and work environments. In addition, in our research extraversion and intellect were poorly and positively correlated with the other-approach, although such relationships were not predicted. In contrast, the predicted relationships of conscientiousness, emotional stability and agreeableness with other-approach were not obtained, although in earlier studies these relationships had occurred, yet they had not been strong^32^. Moreover, in our study, the personality traits were not associated with the other-avoidance factor, even though such relationships were to be expected for nearly all traits from the Big Five model. These avenues will require further research devoted to the relations to the 3 × 2 achievement goal model in sport. In the regression models, our study showed that the personality traits accounted for only a small percentage of the variance (not more than 3%) of achievement goals from the 3 × 2 model in sport. In the light of the discussion regarding to what extent personality is a similar construct to goal orientation/achievements^20,53,54^, the low correlations obtained in our study between these variables support the assumption that the mentioned constructs are rather not redundant. It is even possible that the relationships between the personality traits and goals in sport are mediated by other variables. On another note, maybe achievement goals in sport may rather stem from a favorable developmental context, such as an appropriate motivational setting in the sports environment^55^ than the personality traits themselves. Another factor contributing to lower correlations in groups of athletes may also be their selection in view of certain personality traits. In addition, we also analyzed the relationships between the personality traits and achievement goals in groups of men and women, as well as among high-performance and recreational athletes. Nonetheless, these relationships were also relatively weak, generally not exceeding 0.20, and they did not differ considerably across the groups. This issue requires further research.

The present study is not free from limitations. The study was conducted on relatively young people; it is therefore worth extending the study to athletes of different ages. Performing validation on one group of athletes is also a certain limitation, hence it is worth verifying the validity of the construct on other groups of professional and recreational athletes. In further research and analysis of the Polish version of the 3 × 2 AGQ-S questionnaire it is also worthwhile to compare team and individual athletes with regard to achievement goals (the 3 × 2 model). Moreover, it is vital to expand the study with the testing of convergent validity, so as to evaluate the relationships of the scale with other tools measuring similar constructs. In addition, it is worth extending the validation study to include relations of the 3 × 2 AGQ-S with tools to measure other constructs that are theoretically important from the perspective of the questionnaire under investigation. It is essential to present various measures of the criterion validity of the scale by assessing the relationships of achievement goals in sport with such factors as sport performance. It is also worth analyzing the reliability of the scale using the test–retest method.

In conclusion, the conducted study demonstrates that the Polish version of the 3 × 2 AGQ-S questionnaire shows satisfactory indicators of validity and reliability. It can therefore be used both in scientific research in sport and in the field of sport practice. A significant aspect of this contribution was the extension of validation to high-performance and recreational athletes. In the analysis of measurement invariance, it was demonstrated that individual groups use the 3 × 2 AGQ-S questionnaire in a similar manner. Furthermore, women use the scale similarly to men. Relationships between the Big Five personality traits model and the achievement goals from the 3 × 2 model in sport were also presented. The obtained correlations, however, were not very strong, indicating the need for further investigations into the psychosocial determinants of achievement goals in sport.

## Data Availability

The data generated and/or analyzed during the current study are available from the corresponding author on reasonable request.
